# A new contribution to the classification of stressors affecting nursing professionals

**DOI:** 10.1590/1518-8345.1240.2895

**Published:** 2017-05-22

**Authors:** Jesús Cremades Puerto, Loreto Maciá Soler, Maria José López Montesinos, Azucena Pedraz Marcos, Víctor Manuel González Chorda

**Affiliations:** 1 MSc, RN, Hospital General Universitario "Virgen de la Salud", Elda, Alicante, Spain.; 2 PhD, Professor, Universidad de Alicante, Alicante, Spain.; 3 PhD, Professor, Universidad de Murcia, Murcia, Spain.; 4 PhD, Professor, Universidad Autónoma de Madrid, Madrid, Spain.; 5 PhD, Professor, Universidad de Jaume I, Castellón, Spain.

**Keywords:** Burnout, Professional, Nursing Staff, Hospital, Hospital Units.

## Abstract

**Objective:**

to identify and classify the most important occupational stressors affecting nursing professionals in the medical units within a hospital.

**Method:**

quantitative-qualitative, descriptive and prospective study performed with Delphi technique in the medical units of a general university hospital, with a sample of 30 nursing professionals.

**Results:**

the stressors were work overload, frequent interruptions in the accomplishment of their tasks, night working, simultaneity of performing different tasks, not having enough time to give emotional support to the patient or lack of time for some patients who need it, among others.

**Conclusion:**

the most consensual stressors were ranked as work overload, frequent interruptions in the accomplishment of their tasks, night working and, finally, simultaneity of performing different tasks. These results can be used as a tool in the clinical management of hospital units, aiming to improve the quality of life of nursing professionals, organizational models and, in addition, continuous improvement in clinical treatment.

## Introduction

There is an increasing interest in recent years on the study of work stress^1^ ^-^ ^3^ and the stressors in the work place due to their impact on the health of workers^3^ ^-^ ^8^. Among the negative effects of work stress on the individual, several disorders can be mentioned both physical and psychological or behavioral. These, in turn, can lead to corporate problems, such as increased work absenteeism, reduced quality of work, reduced productivity, job dissatisfaction, work-related accidents, frequent changes of job and abandonment of the profession^7^ ^-^ ^11^, with possible undesirable effects on clinical management and patient care. In the sphere of Health Sciences, according to current studies such as RN4CAST^12^, nursing is considered a stressful profession.

In general, the identification of the main occupational stressors in nursing is related to specific units or contexts, such as Intensive Care Units (ICU), Emergency Services, Specialized Services or Primary Care. However, in medical units of general hospitals, there has been an increasing interest in finding out the specific occupational stressors of these units^13^. It can be stated that there are currently several levels of stress exposure depending on the different medical specialties, areas or services of the hospital, and there is also no international consensus on the choice of method for stress assessment^14^.

In previous related studies in other hospitals, it was observed that the main stressors are related to physical environment, demands of the work itself, work content, performance of the job, components of the organizational structure or work environment^15^.

Stress is considered as an emerging occupational pathology, with a special incidence on the services sector and a greater risk in activities that require personalized dedication^16^. It is understood as a stress factor or stressor, any stimulus capable of eliciting a stress response, with stress being the same response or reaction to a stressor^17^. To reinforce this theoretical model, there is the continuous contribution of several authors, from W. Cannon to JM. Peiró, passing by H. Seyle, Lazarus and Folkman or Ivansevich and Matteson, among others. These are based both on the socio-cognitive theory of the ego, the theory of social interchange and the organizational theory, as well as on the structural theory, in which a concept is developed from a physical point of view to a more holistic model, including psychological and social factors, and internal and external agents^18^ ^-^ ^19^.

Currently, since the collapse of one of the world's largest investment banks, Lehman Brothers Holdings Inc. in 2008, which triggered the current economic slowdown in the industrial world, it has been observed the intensification of occupational stress. Thus, it seems relevant conducting related research, which may be important in the clinical management. 

The general objective of the study is to identify and classify the main occupational stressors affecting the Nursing Professionals (NP) of the hospital medical units. 

## Method

Quantitative-qualitative, descriptive and prospective study developed with Delphi methodology. The Delphi technique is validated as a reliable consensus tool used in studies that require consensus from experts^20^. 

The central element of this study is the University General Hospital of Murcia, with public management and 144 beds in the study units. The hospitalization units incorporated in the study were Medical Units of Internal Medicine, Cardiology, Pulmonology, Rheumatology, Neurology, Nephrology, Allergology, Gastroenterology and Endocrinology, representing a homogeneous sample with similar nursing assignments. 

Surgical Units (Surgery, Orthopedic Surgery and Traumatology, Urology) and Special Units were excluded, as well as Hemodialysis, Emergency and Intensive Care Unit (ICU). 

The study was conducted from December 2012 to April 2013. 

This is an accidental sampling and participants are nursing professionals (NP) who work in the medical units mentioned above and meet the inclusion criteria: 

### Selection criteria for their inclusion:

Nurses who perform clinical nursing in the units mentioned before, for at least 3 years in the medical units and were active during the study. 

### Exclusion criteria

Nurses less than 5 years away to reach retirement; pregnant nurses; staff who did not voluntarily accept to participate in the study.

### Losses

Nurses who were absent from the job during the study due to medical leave; refusal to continue participating at any time. 

Data collection was performed in three stages. The procedure to obtain the answers was carried out by e-mail or mail (sealed envelope), according to the participant's preference. The stages were as follows: 

### First stage

After asking the supervisor of each unit what NP met the inclusion criteria of this study, visits were carried out to the hospital sectors included in the study, requesting the participation to all NP. Among those participants who showed interest, it was evaluated for selection: to meet the inclusion criteria, provide the requested personal data to the research team and accept their status as participants at that moment. Personal data were not disclosed in any document, with the identity of each participant being represented by a number. To those who agreed to participate, it was sent by e-mail or mail in a sealed envelope (according to the participant's choice, to favor their participation), a list of stressors obtained after a literature review according to the classifiers^15^; the definition of each headings^21^ (stressors of the physical environment, demands of the work itself, content of the job or characteristics of the tasks to be performed, job duties, interpersonal and group relationships, career development, new technologies, components of the organizational structure or work environment, and relationship between work and other spheres of life); a sheet with information and instructions, and a working sheet divided into two parts: 

Firstly, a document with empty writing spaces was elaborated within each of the headings of the chosen classifier of stressor^21^, so that, in each section, the participants filled with the stressors that they considered as most important. The second part consisted of a new writing space in order to contribute with comments. Secondary variables (age, sex, level of education, hospital, hospital unit, length of service in the medical units, work schedule, and ratio of nurses per patient) were also collected. It was given a period of three weeks to answer the forms, and two weeks to data collection and analysis, preparation of a new form and participants' rest. 

After obtaining the stressors, a list was drawn up grouping them according to the statements^21^. 

Subsequently, a new form with 108 stressors mentioned by the participants was prepared for their assessment on a scale of 1 to 5 (from no stress to much stress).

### Second stage

A form with 108 stressors was given to each participant for their assessment. 

After completing and collecting all the forms, the analysis of the results (mean and median) was performed.

The 27 stressors with the highest scores were selected, which were the most prominent, showing a good difference, and a new form was prepared. 

### Third stage

Participants were given the form obtained in the previous stage to find out the 10 prioritized stressors ranked in a decreasing scale of stress.

For the analysis of the data of the quantitative assessments performed by the participants, mean and median were calculated for each item of all questionnaires of all stages. The qualitative variables were analyzed based on the percentage of their frequencies.

Statistical analysis was performed using PASW (*Predictive Analytics SoftWare* ) Statistics 18. For the analysis, it was previously established that a mean equal to or greater than 2 and/or a median equal to or greater than 3 should be considered as consensus. Thus, the items were reduced to obtain the most frequent and stressful occupational stressors for the NP. 

The principles of the most recent version of the Declaration of Helsinki (Seoul, 2008), Good Clinical Practices of the European Union and the Organic Law 15/1999, of 13 December, on the Protection of Personal Data were met. The Administration of the Hospital Center authorized the project. The working group met the strict criteria regarding for NP sampling, data storage, sharing and access in order to safeguard their security, privacy and confidentiality.

## Results

The sample is mostly female (83.33%) and there is no participant in the age range of less than 30 years in the sample. Participants from 30 to 39 years are 83.33% of the sample; from 40 to 49 years, 8.33%; and from 50 to 59 years, 8.33%. There are no participants over 59 years of age. 

Regarding the level of training, 91.67% have a degree in Nursing and the rest, besides being graduated, has a master's degree in Nursing Sciences. 

Of the participants, 83.33% worked on a shift schedule, compared to 16.67% who did not work at night, and only one participant worked part time. The ratio of patients per NP is identical in all hospital shifts: 8-10 patients/nurse in the morning shift, 12 patients/nurse in the afternoon shift and 18 patients/nurse in the night shift. 

### First stage

Thirty NP met the inclusion criteria, but the total number of NP who agreed to participate in the study anonymously and voluntarily reached 29 NP in the medical units. Of the 29 documents distributed in the first consignment, 12 NP replied, representing a response rate of 41.38%. The ratio of NP per unit is shown in Table 1.

**Table 1:** List of nursing professionals by Hospital Medical Unit who participated in the study. Murcia, Region of Murcia, Spain, 2013

Hospital Medical Units	Total NP*	NP who agreed**^†^**	RR**^‡^**
Unit 1 (Internal Medicine)	10	10	70% (n=7)
Unit 2 (Neurology, Gastroenterology and Nephrology)	10	9	22.22% (n=2)
Unit 3 (Cardiology, Pulmonology and Neurology)	10	10	30% (n=3)
Total	30	29	41.38% (n=12)

*Nursing professionals who met the inclusion and exclusion criteria †Nursing professionals who agreed to participate in the study ‡Response rate

In total, 108 stressors were identified among the nursing professionals in the Medical Units.

### Second stage

Of the 12 professionals who initially participated, 10 replied, representing a response rate of 83.33%. The analysis of the responses resulted in a list with 27 consensual stressors, which composed the items of the form for the next stage. 

### Third Stage

The total number in the third stage, which turned out to be the last due to the high level of consensus, began with 10 participants, of whom 7 replied. Therefore, the response rate was 70%. The results of the stage that turned out to be the last one due to the high consensus level are shown in Table 2.

**Table 2:** Final results of consensual occupational stressors. Murcia, Region of Murcia, Spain, 2013

Responses	Number of entries (Frequency)	%	Mean	Median
Work overload	7	100%	8.29	10
Frequent interruptions in the accomplishment of their tasks	6	85.71%	8.67	9
Performing different tasks simultaneously	5	71.42%	6.6	7
Night working	4	57.14%	7.5	8
Not having enough time to give emotional support to the patient	4	57.14%	6.25	6
Lack of time for some patients who need it	4	57.14%	4.25	3.5
Consequences of my mistakes for the patient	4	57.14%	3.5	2.5
Having the feeling that at the end of their work there remain pending tasks	4	57.14%	1.75	1.5
Not being able to locate the doctor when necessary	3	42.85%	6.67	7
Performing activities beyond my professional duties	3	42.85%	5.67	7
Witnessing the death of young people	3	42.85%	5.33	6

## Discussion

The inauguration of the hospital took place in January 2005, which evidences the length of service of participants. While some have no more than 3 years of service in the same unit, others have 3 or more years of service in the same hospital medical units, or in the same hospital. Therefore, they have experience in working with these particular types of patients.

In qualitative studies with Delphi methodology, the greater the number of participants, the better the conclusions obtained^22^ and, it has been proven in studies^23^ ^-^ ^24^ that a minimum of 10 to 18 interviewed participants is sufficient for the results to be valid. Due to the high level of consensus in the last stage, with two less people, the results may be considered valid. There is a vast literature addressing the stressors that affect NP in different units, but the presence of the stressors in the medical units detected in this study are scarce in the literature^8^ ^,^ ^13^ ^-^ ^14^.

According to the classifier considered^21^, it was observed that the consensual stressors in nursing are associated with the demands of the work itself, attributions of the job, performance of the job, group relations and relations between work and other aspects of life.

The analysis of the results shows that the stressors with the greatest consensus and highest scores among the NP were those related to organizational problems. Therefore, the results of this study corroborate other studies that presented organizational problems as their results^12^ ^,^ ^25^ ^-^ ^28^.

When these results are compared with those of a previous study^15^, it is observed that they are coincident in terms of organizational problems. Both show as stressors work overload; frequent interruptions in the accomplishment of their tasks, lack of time for some patients who need it, consequences of my mistakes for the patient and, having the feeling that at the end of their work there remain pending tasks. The results obtained differ from other studies depending on the unit or service in which they were carried out. Although they coincide in relation to work overload, some associate stress to the processes of death and suffering^29^ ^-^ ^30^. 

This study has as limitation a very small sample, and therefore, the sample size should be enlarged, based on studies with quantitative methodology that allow inferring and correlating with other studies related to the models of organizational management. In addition, there is a considerable lack of similar studies at international level so that the results on the stressors can be compared in a specific way. It was found a study carried out in another Pediatrics unit, which states that NP find it difficult to provide quality care with limited resources, lack of support and having to assume various responsibilities^31^. However, there are other studies that point out the importance of stressors such as work overload, emotional demands or the conflicting relationship between doctors and nurses^32^ ^-^ ^33^.

The geographic region of this study is restricted to an autonomous community, whereas it would be desirable to increase the studies in different autonomous communities aiming at making comparisons and carrying out improvement actions.

## Conclusion

It can be stated that the main stressors, ordered from the highest to the lowest consensus level, are the following: work overload, frequent interruptions in the accomplishment of their tasks, performing different tasks simultaneously, night working and not having enough time to give emotional support to the patient.

Knowing and valuating all these stressors, from the perspective of nursing professionals, can contribute towards improvements in the clinical management of any hospital unit aiming at increasing the efficiency of the organization and, consequently, the quality of life in the work environment of these workers. It is important to continue investigating and adding solutions to the health professionals of different categories and with different characteristics, that is, it is necessary to design and carry out interventions that allow performing experimental studies on work stress, leading to stronger improvement proposals.
